# Candida auris in a Tertiary Care Hospital in Oman: A Five-Year Study of Epidemiology, Clinical Characteristics, and Antifungal Susceptibility

**DOI:** 10.7759/cureus.109946

**Published:** 2026-05-30

**Authors:** Ahmed Al Mamari, Mubarak Al Yaqoobi, Amal Al Jabri

**Affiliations:** 1 Central Public Health Laboratory, Center for Disease Control and Prevention, Ministry of Health, Muscat, OMN; 2 Microbiology Department, Khoula Hospital, Ministry of Health, Muscat, OMN; 3 Infection Control Department, Khoula Hospital, Ministry of Health, Muscat, OMN

**Keywords:** candida auris, candidemia, colonization, hospital infection, oman

## Abstract

Background

*Candida auris* (*C. auris*)* *is an emerging multidrug-resistant pathogen known for causing persistent colonization, invasive infections, and healthcare-associated outbreaks. Its ability to survive in hospital environments, resist common antifungals, and affect critically ill patients makes it a major global health concern. Understanding local epidemiology and susceptibility patterns is essential to guide prevention and management strategies.

Objective

This study aimed to describe the temporal distribution and antifungal susceptibility patterns of *C. auris* at a tertiary care hospital in Oman (2017-2021) and compare clinical characteristics, risk factors, and outcomes between colonized and infected patients, including candidemia and non‑candidemia subgroups.

Methods

This retrospective, single‑center study included all patients with at least one *C. auris-*positive culture at Khoula Hospital, a tertiary care hospital in Oman (2017-2021). Species identification and susceptibility testing were performed using the VITEK® 2 system (bioMérieux, Marcy-l'Étoile, France) and confirmed at the Central Public Health Laboratories. Clinical and epidemiologic data were extracted from electronic medical records. Patients were classified as colonized or infected based on clinical and microbiologic findings, and the infected group was further subclassified into candidemia and non-candidemia subgroups. Statistical comparisons used Mann-Whitney U, chi-square, or Fisher's exact tests, and odds ratios (OR) with 95% confidence intervals (CI) were calculated for categorical variables, with significance at p < 0.05.

Results

A total of 129 patients (130 isolates) were identified. Cases peaked in 2019 and declined thereafter. Of all patients, 51 (39.5%) had confirmed infection, and 78 (60.5%) were colonized. Candidemia was the predominant invasive presentation (39/51, 76.5%). Infected patients had longer hospitalization (median, 73 versus 57.5 days;* *p = 0.027) and higher central venous catheter use (74.5% versus 56.4%; OR, 2.25; 95% CI, 1.04-4.89; p = 0.037) than colonized individuals. Crude mortality was higher in infected than colonized patients (43.1% versus 28.2%, p = 0.080). Among infected patients, candidemia was associated with greater mechanical ventilation use (94.9% versus 66.7%; OR, 9.25; 95% CI, 1.44-59.51; p = 0.008) and central-line utilization (82.1% versus 50.0%; OR, 4.57; 95% CI, 1.13-18.47; p = 0.026). Crude mortality was higher in candidemia than non-candidemia infections (48.7% versus 25.0%, p = 0.14). Susceptibility testing showed universal fluconazole resistance (100%), very limited amphotericin B activity (5.2%), intermediate voriconazole susceptibility (44.8%), and preserved echinocandin activity (caspofungin, 96.6%; micafungin, 100%) and flucytosine activity (80.0%).

Conclusions

The findings demonstrate that *C. auris* imposes a considerable burden on hospitalized patients, with marked morbidity in those who develop invasive disease, particularly candidemia. The organism's persistent multidrug resistance, with its capacity for sustained transmission, highlights the need for strengthened infection-control practices and continuous surveillance. Preservation of echinocandin susceptibility supports their role as first-line therapy, while high prevalence of azole and amphotericin B resistance emphasizes the importance of targeted antifungal stewardship and early risk identification to reduce both transmission and adverse clinical outcomes.

## Introduction

*Candida auris* (*C. auris*) has emerged over the past decade as a major multidrug-resistant fungal pathogen of global concern, particularly in healthcare settings [1]. Unlike other *Candida* species, *C. auris* exhibits a marked ability for nosocomial transmission, prolonged environmental persistence, and resistance to multiple antifungal classes, creating substantial challenges for infection prevention and clinical management [2,3]. Since its initial identification, *C. auris* has been associated with both invasive infections, particularly candidemia, and asymptomatic colonization, predominantly among critically ill patients with prolonged hospitalization, intensive care unit (ICU) admission, and indwelling medical devices [4,5].

The epidemiology of *C. auris* is characterized by rapid healthcare-associated outbreaks, especially in tertiary care hospitals and ICUs, highlighting the importance of timely surveillance, effective infection-control practices, and antimicrobial stewardship [6,7]. Colonized patients may serve as reservoirs for ongoing transmission, while invasive infections are associated with considerable morbidity and mortality [8,9]. The identification of patient-related risk factors and local antifungal susceptibility patterns is therefore essential for guiding surveillance strategies and optimizing prevention and management efforts [10,11].

Despite the global recognition of *C. auris* as a healthcare-associated threat, knowledge gaps persist regarding its clinical spectrum and the factors driving progression from colonization to invasive disease [12,13]. Antifungal resistance profiles of *C. auris* exhibit considerable geographic and clade-specific variability, influencing empiric treatment choices and highlighting the necessity of routine susceptibility testing [14]. While echinocandins are frequently recommended as first-line therapy for invasive disease, resistance to azoles and polyenes complicates clinical decision-making, particularly in regions with limited access to comprehensive susceptibility testing [15]. Understanding the local resistance landscape is crucial for optimizing patient outcomes and informing stewardship policies [16].

Within the Middle East and Gulf region, *C. auris *outbreaks have been reported from Saudi Arabia, Kuwait, and the United Arab Emirates, predominantly affecting critically ill patients with significant device exposure and multidrug-resistant profiles [17-19]. Regional isolates have demonstrated particularly high rates of azole and amphotericin B resistance, with echinocandins remaining the primary therapeutic option, consistent with global clade-specific patterns [18,19]. Despite this growing regional burden, published data from Oman remain scarce, with no comprehensive study yet describing the full epidemiologic, clinical, and microbiologic profile of *C. auris* in this setting [20,21].

This study presents a comprehensive five-year retrospective analysis of *C. auris* at a tertiary referral hospital in Oman. The primary objective was to describe the temporal distribution and antifungal susceptibility patterns of *C. auris* from 2017 to 2021. Also, we compared clinical characteristics, risk factors, and outcomes between colonized and infected patients, including candidemia versus non-candidemia subgroups, to further characterize the spectrum of disease and identify factors associated with invasive presentations.

## Materials and methods

Study design

This retrospective study was conducted at Khoula Hospital, a tertiary referral center in Oman, and employed a hospital-wide surveillance approach. All patients with at least one culture positive for *C. auris *identified from any ward or clinical unit within the hospital between January 2017 and December 2021 were eligible for inclusion, with no restriction applied based on ward type or clinical specialty. Initial species identification and antifungal susceptibility testing were performed at Khoula Hospital using the VITEK® 2 (bioMérieux, Marcy-l'Étoile, France) automated system. All isolates were subsequently sent to the Central Public Health Laboratories (CPHL) for confirmatory species identification by matrix-assisted laser desorption/ionization time-of-flight mass spectrometry (MALDI-TOF MS), which serves as the reference standard for *C. auris* identification and confirmation in Oman. Isolates not confirmed as *C. auris* at CPHL were excluded from all analyses.

Antifungal susceptibility testing at CPHL was performed using broth microdilution or equivalent standardized methods in accordance with the reference laboratory's validated protocols. Minimum inhibitory concentrations (MICs) were determined for fluconazole, voriconazole, caspofungin, micafungin, amphotericin B, and flucytosine. MIC values were interpreted using the Centers for Disease Control and Prevention (CDC) tentative breakpoints for *C. auris*, which define resistance thresholds as follows: fluconazole of ≥32 µg/mL, amphotericin B of ≥2 µg/mL, and echinocandins (caspofungin and micafungin) of ≥4 µg/mL. Not all isolates were tested against every antifungal agent due to variations in panel availability and isolate viability at the time of testing; susceptibility results are therefore reported as the number of susceptible out of the total number tested for each agent, expressed as a percentage.

Data collection

Clinical and demographic variables were retrieved retrospectively from the hospital electronic medical record (Al Shifa). For patients with multiple positive samples during the study period, only the first confirmed *C. auris* isolate was included in descriptive and outcome analyses to avoid intra-patient clustering. Samples not confirmed as *C. auris* at the reference laboratory were excluded.

Isolation of *C. auris*

*Candida auris* infection was defined as the isolation of *C. auris* from either (i) a sterile site, including blood or cerebrospinal fluid (CSF), regardless of clinical context, or (ii) a non-sterile site, including urine, the skin, wound, or the respiratory tract, in the presence of clinical impact defined as compatible signs or symptoms of infection (e.g., fever, leukocytosis, and purulent discharge), hemodynamic instability, abnormal inflammatory laboratory markers, and/or the initiation of systemic antifungal therapy by the treating clinician. Positive central venous catheter (CVC) tip cultures were classified as indicative of bloodstream infection when accompanied by clinical criteria for infection, consistent with established definitions for catheter-related bloodstream infection.

*Candida auris* colonization was defined as the isolation of *C. auris* from a non-sterile clinical or surveillance specimen in the complete absence of clinical impact, defined as no attributable signs or symptoms, hemodynamic stability, stable inflammatory laboratory parameters, and no initiation of systemic antifungal therapy. Specimens obtained from external drainage bags or collection devices were classified as colonization unless clinical criteria for infection were independently satisfied. In cases where classification was ambiguous, the clinical team's documented assessment and treatment decision were used as the final arbiter of infection versus colonization status.

Case adjudication

The classification of all patients as colonized or infected was performed based on predefined criteria applied to the available clinical, microbiologic, and therapeutic documentation retrieved from the electronic medical record. For patients with multiple positive *C. auris* cultures during the study period, classification was based on the clinical context at the time of the first confirmed positive isolate. The timeline of antifungal therapy initiation relative to culture positivity was reviewed to ensure that treatment decisions reflected a clinical judgment of active infection rather than empiric or prophylactic administration.

Statistical analysis

Data entry was performed using EpiData version 4.7 (EpiData Association, Odense, Denmark), and all statistical analyses were conducted using IBM SPSS Statistics version 27.0 (IBM Corp., Armonk, NY). Continuous variables were assessed for normality using the Shapiro-Wilk test; as most variables were non-normally distributed, they are reported as median with interquartile range (IQR) and compared between groups using the Mann-Whitney U test. Categorical variables are presented as counts and percentages and were compared using the Pearson chi-square test or Fisher's exact test; the latter is applied when any expected cell count was less than five.

Three primary comparisons were performed: (i) infected versus colonized patients across all cases, (ii) candidemia versus non-candidemia among the infected patients, and (iii) antifungal susceptibility profiles across all tested isolates. Given the exploratory and descriptive nature of this study. Missing data were not imputed; analyses for each variable were performed on available cases only, and denominators are specified where they differ from the total sample size. Odds ratios (OR) with 95% confidence intervals (CI) were calculated for all categorical variables to quantify the strength of association between risk factors and outcomes. All hypothesis tests were two-sided, and statistical significance was defined as p < 0.05.

Ethical approval

The study was approved by the Research Ethics Committee of Khoula Hospital (approval number: PRO102021086). Due to its retrospective design and the use of de-identified data, individual informed consent was waived. Patient confidentiality was strictly maintained throughout the study.

## Results

**Figure 1:**
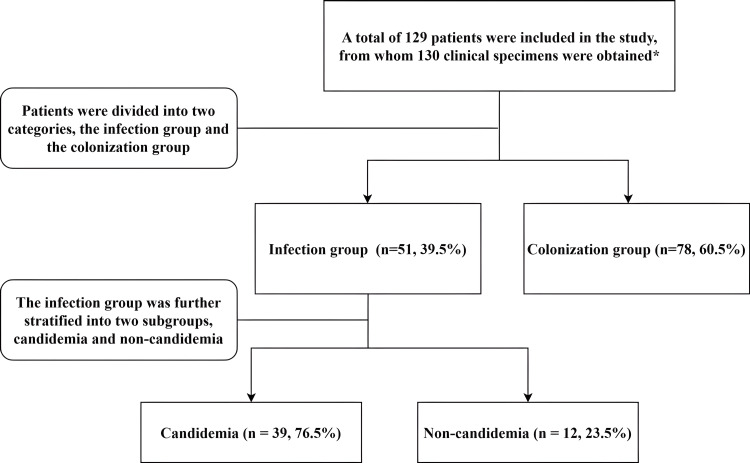
Flow Diagram of Patient Enrolment and Group Allocation. *One patient yielded both peritoneal fluid and blood specimens, which were categorized under blood culture to maintain analytic consistency.

A total of 129 patients met the inclusion criteria and contributed 130 clinical specimens, with one patient providing both peritoneal fluid and blood and classified under blood culture for analytic consistency. Patient enrolment and group allocation are illustrated in Figure 1.

**Figure 2:**
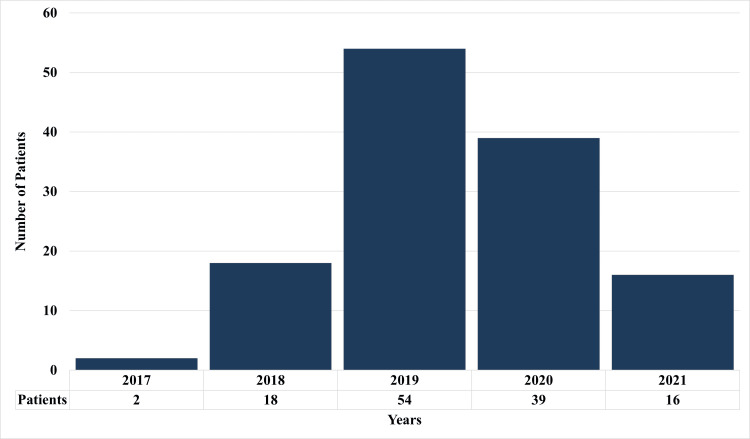
Annual Number of Candida auris Patients (n = 129) (2017-2021).

The distribution of patients over the five-year study period reflects the timing and intensity of the outbreak. Only two (1.5%) patients were identified in 2017, marking the earliest detected cases. The number of patients increased substantially to 18 (14.0%) in 2018 as the outbreak began to expand, followed by a sharp peak in 2019 with 54 (41.9%) patients, representing the height of outbreak activity. In 2020, the number of affected patients declined to 39 (30.2%) and further decreased to 16 (12.4%) in 2021, suggesting either containment measures or waning transmission. These trends provide insight into the temporal dynamics of the outbreak, with the highest burden occurring in 2019. The annual distribution of patients and corresponding specimen contributions is illustrated in Figure 2.

**Figure 3:**
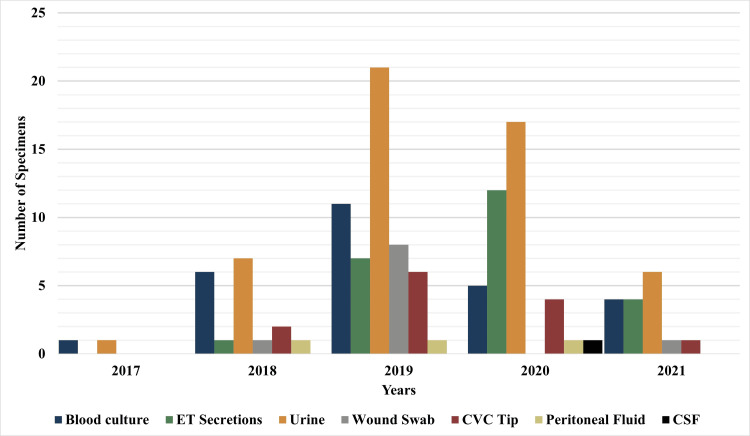
Annual Distribution of Candida auris Isolates by Specimen Type (n = 130) (2017-2021). ET, endotracheal; CVC, central venous catheter; CSF, cerebrospinal fluid

The distribution of specimen types varied over the five-year study period, with blood cultures, urine specimens, and endotracheal secretions representing the most common sources. Blood cultures were most frequent in 2019 (8.5% of 130 isolates) and less common in other years (2017, 0.8%; 2018, 4.6%; 2020, 3.8%; and 2021, 3.1%), reflecting the peak of invasive infections. Urine specimens were also highest in 2019 (16.2%) and 2020 (13.1%), while endotracheal secretions were most frequently collected in 2020 (9.2%) and 2019 (5.4%). Wound swabs and central venous catheter (CVC) tips were collected primarily in 2019 (6.2% and 4.6%, respectively) and CVC tips in 2020 (3.1%), with smaller numbers in other years. Peritoneal fluid and cerebrospinal fluid (CSF) specimens were rare, with only three peritoneal fluid samples (2.3%) and a single CSF sample (0.8%) collected during the study period. Notably, one peritoneal fluid obtained from a drainage bag did not meet the criteria for infection and was therefore classified as colonization (0.8%). Detailed annual distributions of all specimen types are presented in Figure 3.

Among the 129 patients included in the study, 51 met clinical criteria for infection (39.5%), while 78 were classified as colonization (60.5%). Age and sex distributions were similar between the groups, with median ages of 50 years (IQR: 35-71) in the infection group and 57.5 years (IQR: 40.5-70.7) in the colonization group (p= 0.37) and a male predominance in both cohorts (66.7% versus 64.1%, p = 0.76). Most patients were younger than 65 years (68.6% versus 56.4%, p = 0.16). Notably, nationality distribution differed significantly, with a higher proportion of Omani patients among colonized individuals (76.9% versus 58.8%; OR, 0.42; 95% CI, 0.19-0.92; p = 0.029). The length of hospital stay was longer in the infection group, with a median of 73 days (IQR: 44-129) versus 57.5 days (IQR: 30-92.7) for colonized patients (p = 0.027). While overall comorbidities were comparable, neurological disease was more prevalent in the colonization group (62.8% versus 43.1%; OR, 0.45; 95% CI, 0.22-0.92; p = 0.028), whereas other conditions, including diabetes mellitus, hypertension, dyslipidemia, cardiovascular disease, and immunodeficiency, showed no significant differences.

Specimen and risk factor analysis revealed patterns consistent with infection versus colonization. Blood cultures were positive only in the infection group (52.9% versus 0%; OR, 4.25; 95% CI, 2.99-6.03; p < 0.001), as were catheter tips (25.5% versus 0%; OR, 3.05; 95% CI, 2.35-3.96; p < 0.001), while colonized patients more frequently had positive urine samples (55.1% versus 17.6%; OR, 0.17; 95% CI, 0.075-0.41; p < 0.001), endotracheal secretions (30.8% versus 0%; OR, 1.94; 95% CI, 1.61-2.34; p < 0.001), and wound swabs (12.8% versus 0%; OR, 1.75; 95% CI, 1.50-2.05; p = 0.008). Median time to diagnosis tended to be longer in infected patients (30 days versus 22 days, p = 0.062). Most patients in both groups had broad-spectrum antibiotic exposure (96.1% versus 98.7%, p = 0.33), intensive care unit (ICU) admission (92.2% versus 88.5%, p = 0.49), mechanical ventilation (88.2% versus 83.3%, p = 0.44), urinary catheterization (98.0% versus 98.7%, p = 0.76), and wounds (86.3% versus 79.5%, p = 0.32), but central-line use was significantly higher in infected patients (74.5% versus 56.4%; OR, 2.25; 95% CI, 1.04-4.89; p = 0.037). Other interventions, including surgical procedures, external ventricular drain (EVD), ventriculoperitoneal shunt (VPS), chest tubes, total parenteral nutrition (TPN), and SARS-CoV-2 infection, did not differ significantly. Patients were predominantly managed in surgical wards (70.6% versus 56.4%, p = 0.10). Crude mortality was numerically higher in the infection group (43.1% versus 28.2%), although this difference did not reach statistical significance (p = 0.080), as shown in Table 1.

**Table 1:** Demographic, Clinical, and Risk Factor Characteristics of Candida auris Patients (N = 129) by Disease Status: Infection Versus Colonization. Data are presented as N (%) or median (interquartile range {IQR}). Odds ratios (OR) are shown with 95% confidence intervals (CI). Bold values indicate statistically significant differences (p < 0.05). CVC, central venous catheter; ET, endotracheal; CSF, cerebrospinal fluid; EVD, external ventricular drainage; VPS, ventriculoperitoneal shunt; TPN, total parenteral nutrition; ICU, intensive care unit

Variable, N (%)	Infection (N = 51, 39.5%)	Colonization (N = 78, 60.5%)	Odds Ratio (95% CI)	P value
Age, median (IQR)	50 (35-71)	57.5 (40.5-70.75)	-	0.37
Age group				
<65 years	35 (68.6%)	44 (56.4%)	1.69 (0.80-3.55)	0.16
≥65 years	16 (31.4%)	34 (43.6%)		
Sex				
Male	34 (66.7%)	50 (64.1%)	1.12 (0.53-2.35)	0.76
Female	17 (33.3%)	28 (35.9%)		
Nationality				
Omani	30 (58.8%)	60 (76.9%)	0.42 (0.19-0.92)	0.029
Non-Omani	21 (41.2%)	18 (23.1%)		
Transferred from other hospitals	32 (62.7%)	31 (39.7%)	1.14 (0.55-2.33)	0.72
Length of hospital stay, median (IQR)	73 (44-129)	57.5 (30-92.75)	-	0.027
Comorbidities	30 (58.8%)	52 (66.7%)	0.71 (0.34-1.48)	0.36
Diabetes mellitus	15 (29.4%)	30 (38.5%)	0.67 (0.33-1.41)	0.29
Hypertension	24 (47.1%)	38 (48.7%)	0.94 (0.46-1.90)	0.85
Dyslipidemia	4 (7.8%)	10 (12.8%)	0.58 (0.17-1.96)	0.37
Cardiovascular disease	8 (15.7%)	14 (17.9%)	0.85 (0.33-2.20)	0.73
Neurological disease	22 (43.1%)	49 (62.8%)	0.45 (0.22-0.92)	0.028
Immunodeficiency diseases	2 (3.9%)	2 (2.6%)	1.55 (0.21-11.38)	0.66
Specimen				
Blood culture	27 (52.9%)	0 (0%)	4.25 (2.99-6.03)	<0.001
ET secretions	0 (0.0%)	24 (30.8%)	1.94 (1.61-2.34)	<0.001
Urine	9 (17.6%)	43 (55.1%)	0.17 (0.075-0.41)	<0.001
Wound swab	0 (0%)	10 (12.8%)	1.75 (1.50-2.05)	0.008
CVC tip	13 (25.5%)	0 (0%)	3.05 (2.35-3.96)	<0.001
CSF	1 (2.0%)	0 (0%)	2.56 (2.06-3.18)	0.21
Peritoneal fluid	2 (3.9%)	1 (1.3%)	3.14 (0.27-35.59)	0.33
Days to diagnosis, median (IQR)	30 (17-51)	22 (13-38.25)	-	0.062
Risk factor/exposure				
Broad-spectrum antibiotics	49 (96.1%)	77 (98.7%)	0.32 (0.03-3.60)	0.33
ICU admission	47 (92.2%)	69 (88.5%)	1.53 (0.45-5.30)	0.49
Mechanical ventilation	45 (88.2%)	65 (83.3%)	1.50 (0.53-4.24)	0.44
Surgical intervention	35 (68.6%)	45 (56.3%)	1.60 (0.76-3.37)	0.21
Central line	38 (74.5%)	44 (56.4%)	2.25 (1.04-4.89)	0.037
EVD	9 (17.6%)	14 (17.9%)	0.98 (0.39-2.47)	0.96
VPS	4 (7.8%)	4 (5.1%)	1.57 (0.38-6.60)	0.53
Chest tube	2 (3.9%)	1 (1.3%)	3.14 (0.28-35.60)	0.33
Urinary catheter	50 (98.0%)	77 (98.7%)	0.65 (0.04-10.62)	0.76
TPN	6 (11.8%)	5 (6.4%)	1.95 (0.56-6.80)	0.28
Wounds	44 (86.3%)	62 (79.5%)	1.62 (0.62-4.37)	0.32
SARS-CoV-2	4 (7.8%)	9 (11.5%)	1.53 (0.45-5.27)	0.49
Ward				
Surgical	36 (70.6%)	44 (56.4%)	1.86 (0.88-3.93)	0.10
Non-surgical	15 (29.4%)	34 (43.6%)		
Crude mortality	22 (43.1%)	22 (28.2%)	0.52 (0.25-1.09)	0.080

Further analysis of the infection group classified patients into two subpopulations: candidemia and non-candidemia. Among the 51 infected patients, 39 had candidemia (76.5%), while 12 had non-candidemia infections (23.5%). Age and sex distributions were similar between the groups, with a median age of 53 years (IQR: 43-71) in patients with candidemia and 39.5 years (IQR: 27.7-68.7) in patients with non-candidemia (p = 0.19) and an identical male predominance in both subgroups (66.7%). Most patients were younger than 65 years (66.7% versus 75.0%, p = 0.58). Nationality, length of hospital stay, and comorbidities, including diabetes mellitus, hypertension, dyslipidemia, cardiovascular disease, neurological disease, and immunodeficiency, did not differ significantly between patients with candidemia and non-candidemia. Median hospital stay was 73 days (IQR: 45-117) in candidemia cases versus 100 days (IQR: 41.7-172) in non-candidemia cases (p = 0.63).

Significant differences were observed in risk factors and interventions. Mechanical ventilation was more frequent among patients with candidemia (94.9% versus 66.7%; OR, 9.25; 95% CI, 1.44-59.51; p = 0.008), as was central-line use (82.1% versus 50.0%; OR, 4.57; 95% CI, 1.13-18.47; p = 0.026). Ventriculoperitoneal shunts were observed exclusively in patients with non-candidemia (33.3% versus 0%; OR, 5.88; 95% CI, 3.13-11.05; p < 0.001), while external ventricular drains were more common, though not statistically significant, in patients with non-candidemia (33.3% versus 12.8%, p= 0.10). Wounds were present in all patients with non-candidemia and most candidemia cases (100% versus 82.1%, p = 0.11). Broad-spectrum antibiotic exposure, ICU admission, surgical interventions, urinary catheters, TPN, and SARS-CoV-2 infection did not differ significantly. Surgical wards were more commonly involved in non-candidemia cases (91.7% versus 64.1%, p = 0.067). Crude mortality was higher among patients with candidemia (48.7% versus 25.0%), although this difference did not reach statistical significance (p = 0.14), as shown in Table 2.

**Table 2:** Demographic, Clinical, and Risk Factor Characteristics of Candida auris-Infected Patients (N = 51) by Infection Type: Candidemia Versus Non-candidemia. Data are presented as N (%) or median (interquartile range {IQR}). Odds ratios (OR) are shown with 95% confidence intervals (CI). Bold values indicate statistically significant differences (p < 0.05). EVD, external ventricular drainage; VPS, ventriculoperitoneal shunt; TPN, total parenteral nutrition; ICU, intensive care unit

Variable, N (%)	Candidemia (N = 39, 76.5%)	Non-candidemia (N = 12, 23.5%)	Odds Ratio (95% CI)	P value
Age, median (IQR)	53 (43-71)	39.5 (27.7-68.75)	-	0.19
Age group				
<65 years	26 (66.7%)	9 (75.0%)	0.67 (0.15-2.89)	0.58
≥65 years	13 (33.3%)	3 (25.0%)		
Sex				
Male	26 (66.7%)	8 (66.7%)	1.00 (0.25-3.95)	1.00
Female	13 (33.3%)	4 (33.3%)		
Nationality				
Omani	25 (64.1%)	5 (41.7%)	2.50 (0.67-9.37)	0.16
Expatriate	14 (35.9%)	7 (58.3%)		
Transferred from other hospitals	25 (64.1%)	6 (50.0%)	1.79 (0.48-6.60)	0.38
Length of hospital stay, median (IQR)	73 (45-117)	100 (41.75-172)	-	0.63
Comorbidities	23 (59.0%)	7 (58.3%)	1.03 (0.28-3.82)	0.96
Diabetes mellitus (DM)	13 (33.3%)	2 (16.7%)	2.50 (0.48-13.12)	0.26
Hypertension (HTN)	20 (51.3%)	4 (33.3%)	2.11 (0.54-8.16)	0.27
Dyslipidemia (DLP)	4 (10.3%)	0 (0%)	1.34 (1.14-1.59)	0.24
Cardiovascular disease	7 (17.9%)	1 (8.3%)	2.41 (0.27-21.81)	0.42
Neurological disease	15 (38.5%)	7 (58.3%)	0.45 (0.12-1.67)	0.22
Immunodeficiency diseases	2 (5.1%)	0 (0%)	1.32 (1.13-1.55)	1.00
Risk factors/exposure				
Broad-spectrum antibiotics	38 (97.4%)	11 (91.7%)	3.46 (0.20-59.83)	0.36
ICU admission	37 (94.9%)	10 (83.3%)	3.70 (0.46-29.64)	0.19
Mechanical ventilation	37 (94.9%)	8 (66.7%)	9.25 (1.438-59.51)	0.008
Surgical intervention	26 (66.7%)	9 (75.0%)	0.67 (0.15-2.89)	0.58
Central line	32 (82.1%)	6 (50.0%)	4.57 (1.13-18.47)	0.026
EVD	5 (12.8%)	4 (33.3%)	0.29 (0.06-1.35)	0.10
VPS	0 (0%)	4 (33.3%)	5.88 (3.13-11.05)	<0.001
Chest tube	2 (5.1%)	0 (0%)	1.32 (1.13-1.55)	0.42
Urinary catheter	38 (97.4%)	12 (100.0%)	0.760 (0.65-0.89)	0.57
TPN	4 (10.3%)	2 (16.7%)	0.57 (0.09-3.59)	0.54
Wounds	32 (82.1%)	12 (100.0%)	0.72 (0.60-087)	0.11
SARS-CoV-2	4 (10.3%)	0 (0%)	0.75 (0.63-0.88)	0.56
Days to diagnosis, median (IQR)	30 (17-51)	28 (17.5-58.25)	-	0.95
Ward				
Surgical	25 (64.1%)	11 (91.7%)	0.16 (0.02-1.39)	0.067
Non-surgical	14 (35.9%)	1 (8.3%)		
Crude mortality	19 (48.7%)	3 (25.0%)	0.35 (0.08-1.50)	0.14

Antifungal susceptibility testing revealed marked differences in the activity of various agents against the isolates. All 60 isolates tested against fluconazole were resistant, and amphotericin B demonstrated minimal activity, with only 5.2% of 58 isolates being susceptible. Voriconazole showed moderate activity, with 44.8% being susceptible. In contrast, echinocandins exhibited excellent activity, with caspofungin and micafungin demonstrating high susceptibility rates of 96.6% and 100.0%, respectively. Flucytosine also retained substantial activity, with 80% being susceptible. These findings are illustrated in Table 3.

**Table 3:** Antifungal Susceptibility Profile of Candida auris Isolates. Data are presented as n/N (%), where n = number of susceptible isolates and N = total number of isolates tested per antifungal agent. Not all isolates were tested against every antifungal agent due to variations in panel availability and isolate viability at the time of testing. Minimum inhibitory concentrations (MICs) were interpreted using Centers for Disease Control and Prevention (CDC) tentative breakpoints for *Candida auris*.

Antifungal Agent	Susceptible/Tested Isolates (%)
Fluconazole	0/60 (0%)
Voriconazole	26/58 (44.8%)
Caspofungin	57/59 (96.6%)
Micafungin	50/50 (100%)
Amphotericin B	3/58 (5.2%)
Flucytosine	20/25 (80%)

## Discussion

This five-year retrospective study provides a comprehensive overview of *Candida auris* epidemiology, clinical features, and antifungal susceptibility in a tertiary referral hospital in Oman. Our findings demonstrate a substantial burden of colonization, with a smaller but clinically significant proportion of invasive infections, particularly candidemia, among critically ill patients with invasive devices.

A notable temporal trend was observed, with increasing case detection through 2019, representing the peak of outbreak activity during the study period. Although the exact cause cannot be determined, factors such as increased transmission in ICU settings, prolonged hospitalization, invasive device use, and broad-spectrum antimicrobial exposure may have contributed to the surge [22]. Increased awareness and enhanced surveillance during the outbreak period may also have improved case detection [23]. Similar outbreak patterns have been reported in regional studies from Saudi Arabia, where healthcare-associated transmission and environmental persistence played major roles in rapid spread [22].

Following the 2019 peak, a gradual decline in case numbers was observed over the subsequent years. Although the retrospective design limits causal inference, this reduction in cases may have been associated with the implementation and reinforcement of targeted infection prevention measures after an increase in *C. auris* cases was recognized. These measures included active surveillance, contact precautions, the cohorting of colonized patients, and enhanced device-care bundles. Such interventions align with outbreak-control strategies reported in other settings and likely contributed to reduced transmission [24].It is also plausible that the heightened infection-control measures implemented during the COVID-19 pandemic, including enhanced hand hygiene compliance, the universal use of personal protective equipment, visitor restrictions, and stricter patient cohorting, may have contributed to the observed reduction in transmission after 2019 [25].

Our analysis highlights clear distinctions between colonization and invasive disease. Candidemia predominated among infections, often associated with central-line use, mechanical ventilation, and prolonged ICU stay, emphasizing the role of critical-care exposure and invasive devices in facilitating progression from colonization to invasive infection. This pattern aligns with published data describing bloodstream and catheter-associated specimens as more frequently linked to clinically significant infection [26]. In contrast, isolates from respiratory, urine, and wound specimens were more frequently associated with colonization, underscoring their role as potential reservoirs for nosocomial transmission [27].

The antifungal susceptibility profile revealed universal fluconazole resistance, the limited activity of amphotericin B, the intermediate activity of voriconazole, and preserved susceptibility to echinocandins. Flucytosine retained activity in most isolates. These results are consistent with global and regional surveillance, which document high azole resistance with generally preserved echinocandin susceptibility, supporting current recommendations for echinocandin-based empiric therapy in suspected invasive *C. auris* infections [28]. Geographic and clade-specific variability, however, underscores the need for routine local susceptibility testing to guide empiric therapy [29].

Several limitations should be considered when interpreting these findings. The retrospective, single-center design may limit generalizability. In addition, analysis was restricted to the first confirmed *C. auris* isolate per patient to minimize bias from repeated sampling; however, this approach may not fully capture persistent colonization or recurrent isolation over time. The relatively small number of non-candidemia cases may have reduced the statistical power of subgroup analyses. Furthermore, the absence of molecular typing precluded the assessment of transmission dynamics and clade distribution. Nevertheless, this study provides valuable longitudinal data on the epidemiology, clinical characteristics, and antifungal susceptibility patterns of *C. auris* in a tertiary care setting in Oman.

From a clinical and operational perspective, these findings underscore the importance of the active surveillance of high-risk patients, strict adherence to contact precautions, the cohorting of colonized individuals, meticulous environmental cleaning, the optimization of device-care practices with the prompt removal of unnecessary invasive devices, and antifungal stewardship strategies that minimize inappropriate azole exposure while ensuring timely echinocandin therapy when invasive infection is suspected [30].

## Conclusions

This study highlights the substantial burden of *C. auris *colonization and invasive infection in a tertiary care setting, particularly among critically ill patients with prolonged hospitalization and invasive device exposure. The findings demonstrate high resistance to fluconazole and preserved susceptibility to echinocandins, supporting the importance of ongoing antifungal stewardship and infection-control measures. Continued surveillance and early identification remain essential to limit transmission and improve clinical outcomes.
